# Negative pressure breathing: the response of human respiration and circulation to different levels of rarefaction during inspiration

**DOI:** 10.3389/fphys.2024.1443349

**Published:** 2024-11-27

**Authors:** Yury S. Semenov, Julia A. Popova, Petr V. Luzhnov, Artem V. Demin, Tatiana I. Moreva, Evgeny S. Kriushev, Igor A. Nichiporuk, Alexander I. Dyachenko

**Affiliations:** ^1^ Department of Cardiovascular and Respiratory Physiology in Extreme Environments, Institute of Biomedical Problems of the Russian Academy of Sciences, Moscow, Russia; ^2^ Department of Biomedical Engineering, Bauman Moscow State Technical University, Moscow, Russia; ^3^ Department of Clinical and Physiological Research and Expertise, Institute of Biomedical Problems of the Russian Academy of Sciences, Moscow, Russia; ^4^ Department of Molecular and Cellular Biomedicine, Institute of Biomedical Problems of the Russian Academy of Sciences, Moscow, Russia

**Keywords:** negative pressure breathing, circulation, respiration, gas exchange, heart, cerebral circulation

## Abstract

**Introduction:**

Negative pressure breathing is breathing with decreased pressure in the respiratory tract without lowering pressure acting on the torso. We lowered pressure only during inspiration (NPBin). NPBin is used to increase venous return to the heart and is considered as a countermeasure against redistribution of body fluids toward the head during spaceflight. Aims of our study were: to obtain quantitative information on NPBin-induced changes in parameters of circulation and respiration in healthy human at various rarefactions; to identify main processes involved in cardiorespiratory response to NPBin.

**Methods:**

Cardiorespiratory response to 25 min of NPBin were studied, rarefaction ranged from −10 to −25 cmH_2_O. Parameters of systemic, cerebral, and peripheral hemodynamics, as well as respiratory and gas exchange parameters, were measured with non-invasive methods (Finometer, impedance cardiography, rheoencephalography, transcranial Doppler ultrasonography, laser Doppler flowmetry, capillaroscopy). Concentrations of endothelin-1, atrial and brain natriuretic peptides precursors in venous blood, O_2_ and CO_2_ tensions in arterialized capillary blood were measured.

**Results:**

Tidal volume increased, respiratory rate decreased under NPBin with no significant changes in minute ventilation. Group averaged, respiratory rate and tidal volume changed approximately twice relative to their values observed under normal breathing. Despite the decrease in respiratory rate (up to 2-3 breaths/min), the results indicate CO_2_ wash-out. Changes in respiratory and gas exchange parameters were virtually independent of rarefaction level. Synchronous with breathing oscillations of circulatory parameters increased in amplitude under NPBin, while values of the parameters averaged over NPBin period changed little. Amplitude of oscillations in parameters associated with arteries virtually did not change with increasing rarefaction. Inspiration under NPBin reduced left ventricle stroke volume and arterial blood pressure, increased heart rate. Head blood filling decreased during inspiration under NPBin, the decrease increased almost linearly with increasing rarefaction. Parameters returned to their initial values after the end of inspiration. Peak-to-peak amplitude of oscillations under NPBin ranged: stroke volume 17–25 mL, mean arterial pressure 7–9 mmHg, heart rate 14–18 bpm. Peripheral hemodynamics responded to NPBin little.

**Conclusion:**

Changes in stroke volume and central venous pressure during inspiration under NPBin appear to be the major phenomena mediating the effects of NPBin on the cardiorespiratory system.

## 1 Introduction

Negative pressure breathing (NPB) is a breathing mode in which pressure in the airways is maintained below atmospheric pressure (usually by 5-25 cmH_2_O). Negative pressure can be implemented both during the entire respiratory cycle and at its individual phases. Due to technical simplicity, the mode with maintaining reduced pressure in the airways only during inspiration (NPBin) is most often used to solve practical problems.

At the turn of the 19th and the 20th centuries, analogous to NPB or NPBin techniques were proposed to be used in treatment of infectious lung diseases (Kuhn, 1906) and a number of hemodynamic disorders (Bruns, 1911). NPB (Tikhonov et al., 1991) and NPBin (Popova et al., 2022) were proposed to correct undesirable redistribution of body fluids in the cranial direction, which occurs under microgravity conditions. NPBin was suggested as a method of enhancing venous return to the heart to improve the effectiveness of cardiopulmonary resuscitation in hypotonic conditions caused, among other things, by blood loss, and to maintain proper cerebral circulation affected by various unfavorable conditions and pathological processes (Convertino, 2019; Rickards, 2019). Also, similar to NPB or NPBin modes of breathing can be implemented in a wide variety of breathing trainers and breathing exercises.

Despite the wide range of tasks in which NPBin can be used, physiological mechanisms involved in NPBin effect on circulation and breathing were not sufficiently studied since researchers paid most attention to practical applications. Mechanisms of NPB action on circulation, on the contrary, were investigated in detail in works of Baranov et al. (2001), Tikhonov et al. (2003). However, a direct transfer of the results to NPBin is impossible due to a different reaction of respiratory regulation systems (under NPB, profound effect on breathing pattern was not noticed, meanwhile, the pattern changes greatly under NPBin). Effects of an additional resistive or elastic load on respiratory system and associated changes in circulation were also studied in great detail (for example, Axen et al., 1983). But the results of these studies cannot be projected onto NPBin due to a different nature of the effects. Under NPBin, the decrease in airway pressure is practically independent of the inspiratory flow; it is not true in case of resistive or elastic load on breathing.

Among the studies on NPBin, only a few have examined the influence of rarefaction level on the magnitude of NPBin effects. This research aims to fill this gap. Expanding knowledge of NPBin effects on respiration and circulation and obtaining the most complete picture of changes occurring simultaneously in different parts of cardiorespiratory system under NPBin were primary goals of this research. Quantitative results of the research will contribute to a better understanding of the mechanisms of interaction between breathing and blood circulation and will help to choose the optimal value of rarefaction for each type of practical application of NPBin.

In our previous research (Semenov et al., 2024) we introduced a method based on coherent averaging for analysis of synchronous with breathing oscillations of circulatory parameters. The method allows studying in detail the changes in parameters that occur during inspiration or whole respiratory cycle and, unlike spectral methods, gives clear and unambiguous results as it conserves phase relationships between oscillations of the parameters and do not involve transition to frequency domain. Using the method, we found that left ventricle stroke volume and arterial blood pressure parameters decreased during inspiration under NPBin with the rarefaction of 20 cmH_2_O, while mean values of circulatory parameters did not change.

As mean values of circulatory parameters changed little under NPBin or did not change at all, analysis of synchronous with breathing oscillations of circulatory parameters was our main tool in this research. Also we analyzed NPBin-induced changes in respiratory and gas exchange parameters as there are strong relations between respiration and circulation.

Aims of this study were: 1) to characterize how the magnitude of physiological reactions to NPBin changes with increasing levels of inspiratory rarefaction, focusing on the integrated response of cardiorespiratory system through simultaneous measurements of multiple parameters; 2) to establish quantitative relationships between rarefaction levels and physiological responses in resting healthy humans, with the practical goal of providing evidence-based guidance for optimal rarefaction selection in clinical and aerospace applications.

## 2 Materials and methods

### 2.1 Subjects

Seven healthy nonsmoking men of average build aged 19–34 years (mean ± SD = 25.7 ± 6.3) volunteered to participate in this study. All volunteers underwent a medical history and physical examination by a physician to ensure that they had no previous or current medical conditions that might preclude their participation. In accordance with the Declaration of Helsinki, written informed consent was obtained from all participants. All experimental procedures were approved by the Bioethical Commission of the Institute of Biomedical Problems of the Russian Academy of Sciences (protocol № 373 of 31.10.2014).

### 2.2 Procedure and measurements

Five of the same series of measurements were performed with each of the volunteers. All series were conducted according to a single cyclogram, the choice of additional rarefaction during inspiration under NPBin was the only difference (−10, −15, −20, −25 cmH_2_O at mouth level in NPBin series, and 0 cmH_2_O in the control series; hereinafter, all pressure values are given relative to atmospheric pressure). Each series consisted of three stages (before NPBin, NPBin, after NPBin), the position of the volunteer during measurements at either stage was lying on the back horizontally. The main part of each series included free breathing for 15 min (“before NPBin” stage), NPBin for 25 min (“NPBin” stage; in the control series, free breathing continued at this stage), free breathing for 15 min (“after NPBin” stage).

During the main part of each series, volunteers breathed atmospheric air through a mouthpiece while wearing a nose clip. Respiratory flow was divided into inspiratory and expiratory flow using a valve box. During NPBin, the inspiratory valve opened and allowed to breath in if the pressure in the inspiratory channel was below atmospheric pressure by a preset value (−10, −15, −20, −25 cmH_2_O). During free breathing, inspiratory valve opened at almost zero pressure difference (no more than 1.5 cmH_2_O). The expiratory valve produced no resistance during free breathing as well as during NPBin. Expiratory flow was sent to the mixing chamber of gas analyzer (Jaeger Oxycon Pro, Viasys Healthcare GmbH, Germany). Pressure in the mouthpiece (Pm) was continuously recorded (MPX5010DP sensor, Freescale Semiconductor, United States).

For each volunteer, the sequence of series was selected randomly. Which would be the level of rarefaction, the volunteer was not informed before the start of the series or during the series as well as after the end of the current series. For each volunteer, series with different rarefaction levels were conducted on different days, the rest between the series was at least a day and no more than 2 weeks. A few days before experimental series, volunteers were acquainted with NPBin. As training, NPBin was given for 5–10 min with a maximum rarefaction level achievable in the device of about −30 cmH_2_O.

Prior to the start of the main part of the series, heart ultrasonography was performed (also in a supine position). Ultrasound stroke volume measurements were used to correct results of stroke volume measurements obtained during the main part of the series by Finometer Pro (Finapres Medical Systems BV, Netherlands). Ultrasonography was performed with SonoSite 180 plus (SonoSite, Inc., United States).

Venous blood was sampled just before the start of “before NPBin” stage and immediately after the end of “after NPBin” stage (i.e., 15–20 min after the end of NPBin). Immediately after sampling, samples were centrifuged, blood plasma was aliquoted, frozen, and stored at −60°C until enzyme immunoassay was performed. Concentrations of atrial natriuretic peptide precursor [NT-proANP (1–98)], brain natriuretic peptide precursor [NT-proBNP (1–76)], and endothelin-1 precursor [BigET-1 (1–38)] were measured in samples. Plasma concentrations of these substances were measured using BIORAD equipment (United States) and commercial reagent test kits from BIOMEDICA: BI-20892, SK-1204, BI-20082H. Atrial and brain natriuretic peptides reflect myocardial status, their concentration increasing in response to atrial (mainly right) and, respectively, ventricle stretching or injury. Endothelin-1 concentration changes when blood vessel wall is damaged or stretched. The effect of reduced intrathoracic pressure under NPBin on the heart and intrathoracic vessels was evaluated from changes in NT-proANP, NT-proBNP, and BigET-1 concentrations.

Also, before the start of “before NPBin” stage and after the end of “after NPBin” stage, a questionnaire was conducted. In the questionnaire, volunteers were asked to assess the perceived level of load at “NPBin” stage on a scale of 0–7 (where 7 is the maximum load that cannot be endured for a long time). On a scale of −3, −2, −1, 0, +1, +2, +3, where 0 is the usual state, it was proposed to assess well-being in general, perceived depth and respiratory rate, and the state from drowsiness (−3) to excitement (+3). On a scale of 0-6 (where 0 is no symptom), volunteers assessed presence and severity of headache, dizziness, squeezing in the chest, feeling of swelling of the face and the head.

Parameters of systemic, cerebral, and peripheral hemodynamics, respiratory and gas exchange parameters were continuously measured throughout the main part of series.

Central hemodynamic parameters were measured using Finometer Pro. This device, using the modified Peňáz method (Wesseling, 1984), non-invasively measures pulse oscillations of blood pressure and determines systolic (SAP), diastolic (DAP), mean (MAP) arterial pressures, and heart rate (HR). Using obtained pressure curve, it estimates left ventricle stroke volume (SV) by Modelflow method (Wesseling et al., 1993; Jansen et al., 2001), then calculates cardiac output (CO) and total peripheral resistance (TPR) from the obtained values. Parameters are measured in each cardiac cycle. Also, baroreflex sensitivity (BRS) was calculated (La Rovere et al., 2008).

Rheocardiography (RCG) was also used for non-invasive measurements of central hemodynamic parameters (Kubicek et al., 1970). A tetrapolar scheme was used, electrodes were placed according to Sramek scheme modified by Pushkar’ (scheme with current electrode displacement) (Tsvetkov, 2010). Following parameters were calculated from RCG signal (RCG signal is the inverted variable component of the impedance modulus; RCG signal is directly proportional to changes in blood volume in the studied area):

RCG A (Ohm) – amplitude of the systolic wave (vertical distance from the point of the beginning of the RCG pulse wave to its systolic maximum); TT (s) – period of isovolumetric contraction (period from the ECG R peak to the moment the RCG pulse wave begins); ET (s) – ventricle ejection period (period from the moment the RCG pulse wave begins to the first minimum of the first derivative of the RCG signal after the systolic maximum); Tsys (s) – systole duration (the sum of TT and ET in the current cardiocycle); Tdia (s) – diastole duration (duration of the current RR interval minus Tsys). Also, 12-lead ECG was recorded with Jaeger Oxycon Pro (Viasys Healthcare GmbH, Germany).

To assess cerebral circulation, we used rheoencephalography (REG) (Bodo, 2010) in frontal-mastoid lead on the left (electrical impedance of the left half of the head was measured); a tetrapolar scheme was used to measure the impedance (Luzhnov et al., 2019). When analyzing REG data, we used the procedure proposed by Sokolova et al. (1987). When analyzing the pulse wave curve of REG signal (inverted variable component of the impedance modulus), five reference points are distinguished (Supplementary Figure S1): the beginning of the pulse wave, the systolic maximum (point A), the first inflection point after the systolic maximum (point B), a point at a distance of 4/5 of the duration of the current RR interval from the beginning of the wave (point S), the end of the pulse wave (matches with the beginning of the next pulse wave). Then four indices characterizing cerebral circulation are calculated from the REG signal values at points A, B, S. The list of indices is given below:

REG A (Ohm) – amplitude of the systolic peak of the REG signal, value of the REG signal at point A; REG V/A (%) – the ratio of the REG signal value at point B to the amplitude of the systolic peak; REG VO (%) – the ratio of the REG signal value at point S to the amplitude of the systolic peak; REG F (Ohm/s) – REG F = (A + B)/T, where A is the value of REG signal at point A, B is the value of REG signal at point B, T is the duration of the current RR interval in seconds; REG BI (Ohm) – basic impedance of REG (very slowly changing component of the impedance modulus).

All values of the REG signal are measured from the basic value set by the beginning of the pulse wave in the current cardiocycle.

The value of REG A is used to evaluate the intensity of arterial blood supply to the studied area. According to REG V/A, peripheral resistance of arterial and arteriolar vessels in the studied area is evaluated (the lower the value, the lower the resistance of vessels). The REG VO value is used to evaluate conditions of blood return from the cerebral venous bed. The increase in REG VO corresponds to the increased hindrance to venous return, the decrease corresponds to relief. Unlike other REG indices, REG VO can take negative values. Volumetric blood flow rate is estimated by REG F. The increase in REG F corresponds to an increase in tissue perfusion.

Rheoencephalography and rheocardiography measurements were performed continuously during the series at 100 kHz using multichannel rheograph (Reo-Spectrum, Neurosoft, Russia) and standard self-adhesive round gel ECG electrodes. RCG A, TT, ET, Tsys, Tdia, REG A, REG V/A, REG VO, REG F were calculated in each cardiocycle. Before calculating, harmonics with frequencies below 0.5 Hz were removed from the signal (both RCG and REG), which made it possible to exclude artifacts caused by respiration.

We also used transcranial Doppler ultrasonography (TCD) to measure blood flow velocity in common carotid (CCA) and middle cerebral (MCA) arteries on the right (Sonicaid Vasoflo 4, Oxford Sonicaid Ltd., United Kingdom). The following parameters were analyzed:

PSV (cm/s) – peak systolic velocity; EDV (cm/s) – end-diastolic velocity; TAM (cm/s) – velocity averaged over the current cardiocycle.

Capillaroscopy and laser Doppler flowmetry (LDF) were used to assess microcirculation. With LDF we measured LDP of the skin microvasculature (Laser-Doppler Perfusion, perf. u.; Leahy et al., 1999). Measurements were performed on the right posterior forearm 3–4 cm above the wrist (LAKK-M, SPE “LAZMA”, Russia).

The linear size of the pericapillary zone (PZ) was measured using a capillaroscope. This parameter reflects the degree of tissue hydration (increasing with the increase in hydration). Capillaries of the nail fold of the right hand fingers were examined (Capillaroscan-1, “Advanced Energy Technologies” Ltd., Russia). In each series, measurements were made for 10–30 capillaries at “before NPBin” stage as well as “after NPBin” stage, and for 30–50 capillaries at “NPBin” stage. For each stage, the arithmetic mean PZ value was calculated from the measured capillary set. Only this average value was used for further processing.

We used Pm data to obtain time characteristics of respiratory pattern including respiratory rate (BF). Respiratory and gas exchange parameters (VTex (L, BTPS), V'E (L/min, BTPS), V'O_2_ (mL/min, STPD), V'CO_2_ (mL/min, STPD), RER) were measured with Jaeger Oxycon Pro (Viasys Healthcare GmbH, Germany) using mixing chamber, all measurements were derived from the expiratory flow.

In addition, a transcutaneous O_2_ and CO_2_ tension monitor (TCM4, Radiometer Medical ApS, Denmark) was used to assess changes in gas exchange (tcpO_2_, tcpCO_2_). The sensor was placed on the chest surface in the left subclavian region. The monitor was also used for pulse oximetric measurements of arterial blood oxygen saturation (SpO_2_); the pulse oximeter probe was placed on one of the fingers of the left hand.

Also, tension of O_2_ and CO_2_ dissolved in arterialized capillary blood (pO_2_ and pCO_2_) were measured to assess changes in gas exchange. Blood samples were taken from the fingers of the left hand twice in each series: at the 13–15 min of “before NPBin” stage and at the 24–25 min of “NPBin” stage. Sample analysis was performed immediately after sampling with ABL80 FLEX (Radiometer Medical ApS, Denmark).

### 2.3 Data analysis

The processing of obtained data was carried out for all of the methods in the same way. The processing of continuous measurement data (Finometer Pro, REG, RCG, LDF, TCM4, TCD) consisted of two parts:

1) calculation of average-over-stage values of the parameters in each volunteer, then their changes calculation (relative to “before NPBin” stage), then statistical analysis of the changes;
2) analysis of respiratory oscillations of the parameters.

Analysis of the results of single-point methods (concentrations of precursors of natriuretic peptides and endothelin, O_2_ and CO_2_ tension in capillary blood, questionnaire) included only calculation of changes in parameters between current stage and “before NPBin” stage. Statistical processing of the results was similar to the processing of average-over-stage values of continuously recorded parameters.

#### 2.3.1 Statistical analysis

**FIGURE 1:**
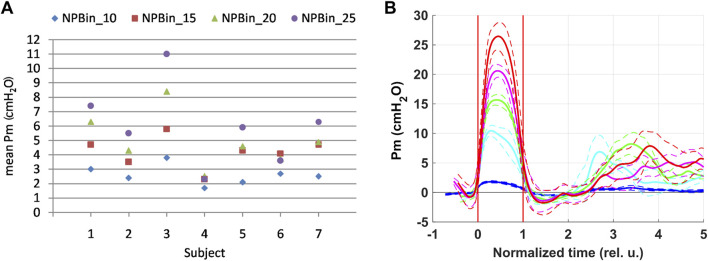
Rarefaction in the mouthpiece (Pm) at “NPBin” stage of experimental series. The rarefaction increases when moving up the vertical axis. **(A)** represents the average Pm values for the entire “NPBin” stage (simple average over the entire recording–without distinguishing inspiration and expiration). **(B)** shows changes in Pm during inspiration under NPBin (curves are averaged over the group of volunteers and over inspirations; at about 2.5 of time normalized to individual duration of inspiration, the next breath began in some volunteers). Colors correspond to series: blue–NPBin_0 (control series, no additional rarefaction), cyan–NPBin_10 (rarefaction during inspiration set to −10 cmH_2_O), green–NPBin_15 (rarefaction during inspiration set to −15 cmH_2_O), magenta–NPBin_20 (rarefaction during inspiration set to −20 cmH_2_O), red–NPBin_25 (rarefaction during inspiration set to −25 cmH_2_O). Solid curve–average Pm changes at “NPBin” stage of each series; dashed curves of the same color shows the boundaries of ±SD interval. Vertical lines mark the edges of inspiration.

Under NPBin, respiratory rate (BF) decreased by 1.5–2 times relative to the free breathing rate, regardless of the level of additional rarefaction during inspiration. Some volunteers showed a profoundly greater decrease in BF: up to 2–3 times per minute. The decrease in BF was caused mainly by appearance or increase in pause between expiration and subsequent inspiration. Such changes in respiratory pattern under NPBin resulted in rarefaction averaged over the entire respiratory cycle or over entire “NPBin” stage being dramatically less than rarefaction during inspiration. Large individual differences in BF changes also resulted in large differences in averaged over “NPBin” stage Pm values (Figure 1).

Individual scattering of averaged over “NPBin” stage Pm values led to the fact that series with different set levels of rarefaction differed little in mean Pm values. When processing average-over-stage parameter values, we combined the data measured in all volunteers of all series with actual NPBin. Thus, when analyzing average-over-stage values, two sets of data were considered: data from the control series and combined data from all series with actual NPBin. We used Wilcoxon rank sum test to detect statistically significant differences between these two datasets, data related to the same stages of different series were compared. Also, in the second set (with actual NPBin), if the test showed significant (*p* < 0.05) differences with the control series, correlations with the averaged over “NPBin” stage Pm value (without distinguishing inspiration and expiration) were analyzed using Spearman’s rank correlation coefficient. Similarly, correlations with the level of rarefaction during inspiration under NPBin were also analyzed. Thus, using statistical analysis, two questions were answered: 1) whether average-over-stage values of the parameters changed in response to NPBin; 2) was there a dependence of the magnitude of changes in the average values on the averaged over “NPBin” stage rarefaction? To minimize the influence of possible individual variations in initial values of physiological parameters on the results of analysis, individual differences in parameters were used as input to the statistical analysis in both cases: the average value of the parameter in the volunteer at the current stage minus the average value of the parameter in the volunteer at “before NPBin” stage of the current series. Unless otherwise specified, descriptive statistics for average-over-stage values in the text below are provided for the combined datasets as follows: M [Q1; Q3], where M is the median calculated over the group of volunteers, Q1 and Q3 are the first and the third quartiles calculated over the dataset.

#### 2.3.2 Respiratory oscillations analysis

Since the rarefaction during inspiration is the determinant in this analysis, and because the rarefaction during inspiration is determined by characteristics of the NPBin valve and is not subject to large individual scattering like the rarefaction averaged over “NPBin” stage, series with different set levels of rarefaction were considered separately.

Respiratory oscillations were analyzed according to the procedure based on the coherent averaging method, the procedure described in detail in Semenov et al. (2024). Respiratory oscillations analysis was not performed for parameters that could not change rapidly (within a second) due to physical characteristics of the measurement method or due to physiological processes affecting the value of the considered parameter, such parameters included, for example, BRS and transcutaneous monitor data.

Briefly, the calculation of respiratory oscillations was performed as follows. Synchronous recordings of a parameter and Pm were cut into “frames” corresponding to discrete respiratory cycles. “Frames” were superimposed on one graph so that the moments of the beginning of inspiration determined from the recording of Pm were matched. Over the obtained cloud of points representing oscillations of the parameter, a smoothing approximating curve was calculated. The curve is “respiratory oscillation”. To average curves over the group of volunteers, the average parameter value was subtracted from the curve, and the time axis was normalized to the individual duration of inspiration (after normalization, the beginning of inspiration along the time axis corresponds to 0, the end of inspiration to 1). After mean value subtraction and time normalization, the curves obtained from individual volunteers were averaged. In all cases, RLOWESS method or, if there were too many points, LOWESS was used to calculate the approximating curve (Cleveland, 1979).

In a sufficiently long recording, non-standard respiratory cycles or artifacts occur from time to time. The “frames” corresponding to them were thrown out of the analysis before superimposing the “frames” on each other. Non-standard respiratory cycles were determined by the duration of the phases of the respiratory cycle. Distributions of phase durations were plotted for the entire stage under analysis. If the duration of inspiration and duration of the entire respiratory cycle were beyond the specified interval (out of the interval from 10th to 90th percentile), the corresponding respiratory cycles were excluded from the analysis.

Before dividing recordings into “frames”, slow drift of parameters during the stage was subtracted from the recordings (by subtracting linear trend line calculated over the entire stage).

LDF parameters are characterized by strong slow non-linear drift, which sometimes exceeds the peak-to-peak amplitude of respiratory oscillations of the parameters. Therefore, the current baseline value of LDP obtained by smoothing the original data by the moving average method with a window of 5 min was subtracted from the recordings before calculating respiratory oscillations.

When analyzing respiratory oscillations, before calculating the curve averaged over the group of volunteers, the direction of individual reactions was assessed. It was the same in all volunteers and reproduced from series to series, which made it possible to calculate the average curve over the group of volunteers.

The significance of the differences in the obtained curves can be roughly estimated using SD. For arbitrary points of a curve or points of different curves, the differences are considered to be significant if they exceed 2 SD.

Curves related to all stages of the control series did not differ within the ±SD range. Moreover, these oscillations virtually coincided with the observed at “before NPBin” and “after NPBin” stages in other series. Therefore, the respiratory oscillation obtained in the control series at “NPBin” stage gives complete information on oscillations at “before NPBin” and “after NPBin” stages of the other series. Figures presented below shows curves only for “NPBin” stage of any of the series.

## 3 Results

### 3.1 Questionnaire

According to the questionnaire results, no perceived changes in well-being were detected under NPBin. Only perceived breathing depth increased significantly (*p* < 0.001) by 1-2 points (2 [1; 2]) under NPBin, no correlation with the level of rarefaction was found. The perceived load level under NPBin was from 1 to 6 points (the extremes were met once per study); regardless of the actual level of rarefaction, the most frequent answers were 3 and 4. Thus, it can be stated that NPBin even in series with rarefaction during inspiration of −25 cm of water column was a fairly easy-to-endure load (roughly corresponding to the description “moderate” or “somewhat hard”). There was no correlation between the perceived load level and the actual level of rarefaction when considering series with actual NPBin (that is, without taking into account the control series).

### 3.2 Breathing pattern

Under NPBin, there was a pronounced pause between expiration and subsequent inspiration (with free breathing, there were no pauses between inhalation and exhalation); duration of inspiration, duration of expiration, and the total duration of the respiratory cycle increased significantly (*p* < 0.05), respectively, BF decreased significantly (up to 2-3 breaths/min in individual volunteers). The duration of inspiration increased from 1.7 [1.6; 2.1] seconds at “before NPBin” stage to 3.0 [2.6; 3.6] seconds under NPBin, the duration of expiration increased from 2.4 [2.1; 3.0] to 3.9 [3.1; 4.9] seconds, the duration of pause from expiration to subsequent inspiration was 1.4 [0.7; 4.7] seconds under NPBin. The duration of the respiratory cycle increased from 5.0 [4.3; 6.0] to 10.0 [8.2; 13.4] seconds, and respiratory rate decreased from 12.1 [10.0; 13.9] to 6.0 [4.5; 7.3] min^−1^. In the vast majority of volunteers, characteristics of breathing pattern returned to initial (before NPBin) values in several respiratory cycles after the end of “NPBin” stage. In some volunteers, pattern changes persisted for several minutes after the end of “NPBin” stage. A significant correlation of changes in the duration of the pause from expiration to subsequent inspiration with the value of the increase in average-over-stage level of rarefaction was found, and, accordingly, a significant correlation was found for both the duration of the respiratory cycle and respiratory rate. With the increase in rarefaction, the duration of the pause and NPBin-induced increase in the duration of the respiratory cycle decreased (*p* = 0.005, ρ = −0.51 and *p* = 0.026, ρ = −0.42, respectively). For increases in durations of inspiration and expiration, no significant correlation with the level of rarefaction was found. For all the parameters considered, we found no significant correlation of the magnitude of changes with the level of rarefaction during inspiration under NPBin.

### 3.3 Respiration and gas exchange

Changes in breathing pattern during the transition from free breathing to NPBin and *vice versa* completed within a few minutes, regardless of the level of rarefaction. Then characteristics of the pattern and parameters of respiration and gas exchange remained stable. All considered parameters, except for V'E, increased significantly (*p* < 0.005) under NPBin. VTex increased from 0.67 [0.59; 0.75] to 1.53 [0.91; 2.08] L, V'O_2_ and V'CO_2_ rose from 235 [217; 245] to 300 [246; 365] mL/min and from 192 [177; 206] to 278 [210; 371] mL/min, respectively. RER increased from 0.81 [0.76; 0.87] to 0.90 [0.83; 0.96]. V'E increased from 7.5 [7; 8] to 10.5 [7; 12] L/min. Respiratory and gas exchange parameters returned to their initial (before NPBin) values within a few minutes after the end of “NPBin” stage. There was no significant correlation of values of the increase in parameters with the level of rarefaction averaged over “NPBin” stage. However, we found a significant correlation of VTex, V'O_2_, and V'CO_2_ with the level of rarefaction during inspiration under NPBin. The increase increased with the level of rarefaction: VTex–ρ = 0.41, *p* = 0.037; V'O_2_ – ρ = 0.43, *p* = 0.027; V'CO_2_ – ρ = 0.49, *p* = 0.011.

### 3.4 Transcutaneous data

Induced by NPBin changes were significant only in tcpCO_2_ (*p* = 0.002); tcpCO_2_ decreased from 44.0 [41.9; 46.3] mmHg during free breathing to 40.4 [32.7; 43.2] mmHg under NPBin. After the end of “NPBin” stage, tcpCO_2_ slowly returned to its initial (before NPBin) value within 5–10 min. We found no correlation of tcpCO_2_ decrease with the level of rarefaction during inspiration under NPBin or with the level of rarefaction averaged over “NPBin” stage.

### 3.5 Capillary blood data

Significant (*p* = 0.013) changes in response to NPBin were observed in pCO_2_ only. CO_2_ tension in arterialized capillary blood decreased from 43 [41; 45] mmHg during free breathing to 41 [34; 44] mmHg under NPBin. We found no correlation of changes with the level of rarefaction during inspiration under NPBin or with the level of rarefaction averaged over “NPBin” stage.

### 3.6 ECG

We found no clinically significant ECG changes under NPBin.

### 3.7 Venous blood data

We found no significant NPBin-induced changes in concentrations of NT-proANP, NT-proBNP, and BigET-1 (*p* > 0.05). Concentrations before NPBin were: NT-proANP – 1.03 [0.90; 1.31] nmol/L, NT-proBNP – 16.9 [10.2; 26.0] pmol/L, BigET-1 – 0.22 [0.18; 0.30] pmol/L. Concentrations after NPBin were: NT-proANP – 1.42 [1.05; 1.82] nmol/L, NT-proBNP – 13.6 [7.3; 19.1] pmol/L, BigET-1 – 0.25 [0.20; 0.35] pmol/L.

### 3.8 Finometer data

Among average-over-stage values, only DAP, MAP, HR, TPR, BRS showed significant (*p* < 0.05) changes in response to NPBin. Although the changes were significant, the magnitude of the changes in DAP, MAP, HR, TPR was extremely small (Table 1; Supplementary Table S1). Changes did not persist after the end of “NPBin” stage. Also, only BRS changes showed a significant correlation (*p* = 0.003, ρ = −0.54) with the level of rarefaction averaged over “NPBin” stage, the decrease in BRS was more pronounced with an increase in the level of rarefaction. There was no correlation between the magnitude of changes in parameters and the level of rarefaction during inspiration under NPBin.

**TABLE 1:** Circulatory parameters under NPBin and in the control series.

NPBin series				
	Average-over-stage values of parameters			Changes in average-over-stage parameter values under NPBin (“NPBin” stage minus “before NPBin” stage)
	Before NPBin	NPBin	After NPBin	
SAP, mmHg	121.6 [113.2; 126.4]	120.7 [111.2; 128.5]	126.5 [121.1; 131.5]	0.5 [−1.4; 2.7]
DAP, mmHg	68.0 [62.8; 72.4]	68.1 [63.4; 72.3]	72.9 [67.9; 73.8]	0.3 [−0.9; 1.8] **
MAP, mmHg	86.5 [81.2; 92.3]	87.1 [80.0; 92.9]	92.0 [86.4; 95.0]	0.5 [−0.9; 2.1] *
HR, bpm	63.9 [59.6; 66.6]	64.3 [59.9; 69.3]	60.6 [57.9; 67.5]	1.4 [−0.8; 3.7] *
SV, mL	81.9 [70.4; 104.0]	86.1 [70.7; 101.2]	84.4 [70.7; 103.9]	0.4 [−2.0; 2.1]
TPR, mmHg∙s/mL	0.93 [0.88; 1.10]	0.94 [0.82; 1.13]	1.05 [0.91; 1.26]	−0.02 [−0.07; 0.03] *
BRS, ms/mmHg	22.0 [17.2; 25.1]	16.7 [14.4; 19.0]	22.7 [16.0; 27.3]	−3.7 [−7.6; −1.6] +
CCA PSV, cm/s	95.2 [79.7; 104.8]	83.8 [74.9; 97.0]	89.2 [80.3; 98.4]	−9.4 [-14.4; 1.2] *
CCA EDV, cm/s	18.4 [14.9; 21.4]	14.6 [10.0; 16.9]	17.4 [15.3; 20.0]	−5.5 [−6.7; −1.5] +
CCA TAM, cm/s	38.0 [32.3; 39.1]	30.9 [27.7; 34.8]	33.9 [30.8; 37.3]	−6.0 [−7.8; −2.2] *
MCA PSV, cm/s	86.6 [77.0; 96.5]	78.3 [67.4; 96.6]	84.6 [71.0; 97.7]	−6.3 [−17.1; 1.1]
MCA EDV, cm/s	37.2 [32.8; 44.9]	30.0 [22.8; 42.2]	36.5 [30.9; 45.5]	−3.2 [−11.5; −0.4] *
MCA TAM, cm/s	53.4 [48.5; 63.6]	44.9 [36.9; 61.7]	51.8 [45.7; 64.6]	−6.1 [−14.4; −0.5]
Control series				
SAP, mmHg	118.2 [116.3; 124.7]	122.0 [119.0; 126.1]	126.9 [124.4; 133.4]	1.8 [1.2; 5.0]
DAP, mmHg	67.8 [64.8; 70.6]	69.7 [66.9; 72.7]	72.2 [68.9; 75.1]	2.1 [1.3; 2.4]
MAP, mmHg	85.7 [83.7; 89.0]	88.6 [87.1; 91.6]	91.8 [90.9; 95.6]	2.8 [1.7; 3.7]
HR, bpm	64.5 [60.4; 71.0]	63.9 [60.0; 70.5]	61.9 [57.7; 69.7]	−1.1 [−2.4; 0.3]
SV, mL	87.6 [76.1; 101.5]	93.4 [75.0; 99.1]	87.7 [74.1; 100.1]	−0.9 [−1.9; 1.9]
TPR, mmHg∙s/mL	0.94 [0.85; 1.00]	0.94 [0.91; 1.06]	1.02 [0.94; 1.10]	0.06 [0.01; 0.09]
BRS, ms/mmHg	19.0 [15.8; 25.8]	19.5 [14.6; 28.1]	20.2 [15.7; 30.7]	1.3 [−0.5; 2.7]
CCA PSV, cm/s	96.4 [91.0; 100.9]	92.1 [90.5; 101.3]	88.8 [81.4; 99.0]	4.1 [−6.4; 9.0]
CCA EDV, cm/s	17.8 [16.4; 22.4]	18.2 [17.8; 21.6]	20.3 [15.2; 24.4]	0.8 [−0.6; 2.1]
CCA TAM, cm/s	38.7 [35.0; 42.9]	36.8 [35.7; 41.9]	38.0 [33.6; 38.9]	1.4 [−2.8; 2.3]
MCA PSV, cm/s	94.6 [75.6; 115.6]	92.5 [75.6; 98.9]	89.6 [79.0; 97.5]	−3.8 [−7.4; −0.5]
MCA EDV, cm/s	34.6 [30.6; 50.1]	35.5 [33.9; 50.3]	34.0 [32.8; 50.8]	2.5 [−4.1; 4.5]
MCA TAM, cm/s	49.7 [47.2; 74.1]	53.1 [49.2; 69.6]	51.3 [48.3; 70.4]	−0.1 [−4.6; 4.0]

The data are presented as M [Q1; Q3], where M is the median, Q1 and Q3 are the first and the third quartiles, values are calculated over the group of volunteers. Signs denote: * - *p* < 0.05, ** - *p* < 0.01, + - *p* < 0.001. *p*-values were obtained by comparing the same stages of the control series and combined data from NPBin series, using the Wilcoxon rank sum test. When calculating the criterion, we used values of changes in parameters relative to their value at “before NPBin” stage. Please refer to “Materials and Methods” section for further details.

**FIGURE 2:**
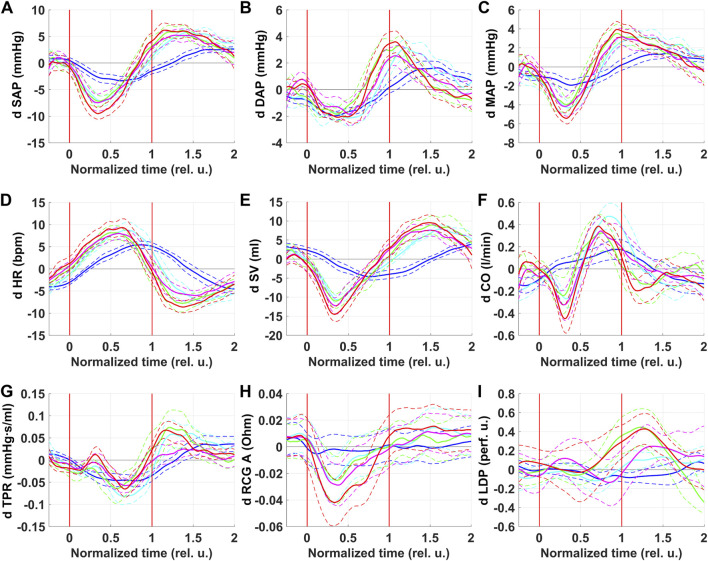
Respiratory oscillations of hemodynamic parameters. SAP–systolic arterial pressure **(A)**, DAP–diastolic arterial pressure **(B)**, MAP–mean arterial pressure **(C)**, HR–heart rate **(D)**, SV–left ventricle stroke volume **(E)**, CO–cardiac output **(F)**, TPR–total peripheral resistance **(G)**, RCG A—systolic maximum of RCG signal **(H)**, LDP–laser Doppler perfusion **(I)**. Colors correspond to series: blue–NPBin_0 (control series, no additional rarefaction), cyan–NPBin_10 (rarefaction during inspiration set to −10 cmH_2_O), green–NPBin_15 (rarefaction during inspiration set to −15 cmH_2_O), magenta–NPBin_20 (rarefaction during inspiration set to −20 cmH_2_O), red–NPBin_25 (rarefaction during inspiration set to −25 cmH_2_O). Solid curve–changes of parameters at “NPBin” stage of each series averaged over breathing cycles and over the group of volunteers; dashed curves of the same color shows the boundaries of ±SD interval. Vertical lines mark the edges of inspiration. Prefix “d” denotes that the average value of the parameter was subtracted before the calculating of the oscillation (i.e., only the variable component was analyzed).

Respiratory oscillations were observed for all central hemodynamic parameters. The results of respiratory oscillations analysis of central hemodynamic parameters are shown in Figure 2A–H.

For all parameters, curves corresponding to NPBin_10, NPBin_15, NPBin_20, and NPBin_25 series (hereinafter, number in the series name denotes the level of rarefaction during inspiration under NPBin in cmH_2_O) virtually matched but differed profoundly from the curve obtained in NPBin_0 series. Peak-to-peak amplitude of oscillations greatly exceeded the changes in the average-over-stage values of corresponding parameters.

SV, SAP, MAP, CO were the first to respond to a decrease in airway pressure during inspiration under NPBin (parameters decreased, reached a minimum in the first half of inspiration). A little later, DAP reached the minimum. HR began to rise immediately after the start of inspiration, but it reached the maximum only at the beginning of the second half of inspiration. The TPR dynamics were multiphase, but decline prevailed, TPR reached its minimum in the second half of inspiration. CO showed two-phase dynamics during inspiration: it decreased in the first half of inspiration and increased above the initial value in the second half, the moment of reaching the maximum approximately corresponded to the minimum of TPR and the maximum of HR. Parameters returned to their initial values after the second half of inspiration, some of them through overshoot. The effect of NPBin on respiratory oscillations of central hemodynamic parameters did not persist after the end of “NPBin” stage.

For all parameters, a simple sinusoid-like oscillation was observed in the control series (NPBin_0). One extremum was approximately at the middle of inspiration, the opposite one was about the middle or the end of expiration. The direction of changes was the same as that observed in series with actual NPBin. The oscillation range in series with actual NPBin was profoundly larger than the range in the control series.

Primarily responded to NPBin parameters were that the first to reach the extremum: SV and SAP. MAP is derived from SAP and DAP, so its dynamics are a mixture of SAP and DAP changes. HR changes lagged slightly behind SV changes. SV, reaching minimum in the first half of inspiration, and HR, reaching maximum in the second half of inspiration, determined two-phase dynamics of CO during inspiration (CO is the product of SV and HR). The complex response of TPR to inspiration is likely associated with a large number of oppositely directed regulatory influences.

### 3.9 RCG

Among RCG parameters, significant differences in changes of average-over-stage values between NPBin series and the control series were observed only for RCG A (it decreased from 0.229 [0.185; 0.243] Ohm during free breathing to 0.211 [0.170; 0.221] Ohm under NPBin, *p* = 0.011). In the control series, RCG A also decreased during the series, but the decrease was not as pronounced as under NPBin (Supplementary Table S1). We found no correlation of NPBin-induced changes in RCG A with the level of rarefaction averaged over “NPBin” stage or with the level of rarefaction during inspiration under NPBin.

RCG A decreased during inspiration (Figure 2H). There were no respiratory oscillations of RCG A in the control series (NPBin_0). The decrease in RCG A reflects a decrease in stroke volume.

Period of isovolumetric contraction (TT) rose slightly during inspiration. The increase was about twice as large under NPBin regardless of the level of rarefaction. Peak-to-peak amplitude of TT respiratory oscillations was about 5 ms under NPBin. Ventricle ejection period (ET) decreased during inspiration by 10–20 ms and rose above its initial value by the same number during expiration. Although the decrease in ET was approximately 1.5 times greater under NPBin than in the control series, the curves obtained in all series did not differ within ±SD range. In NPBin series, the rarefaction level did not affect the decrease in ET. Direction of changes in TT or ET under NPBin was the same as during free breathing. Most likely, changes in TT and ET during breathing cycle are associated with direct mechanical action (changes in intrathoracic pressure and pressures in ventricles and main arteries during breathing shift in time the moments, when pressure equilibrium occurs on heart valves).

Duration of systole (Tsys) is the sum of TT and ET and reflects their changes. Tsys decreased during inspiration and increased during expiration, magnitudes of the decrease and subsequent increase were the same in all series and were almost entirely determined by ET changes. Duration of diastole (Tdia) decreased during inspiration and increased above its initial value by the same number during expiration. Peak-to-peak amplitude of respiratory oscillations under NPBin was about 1.5 times greater than during free breathing, and it was about 170 ms regardless of the level of rarefaction during inspiration under NPBin. Respiratory oscillations of HR were caused mainly by respiratory oscillations in Tdia since Tsys oscillated little.

The effect of NPBin on respiratory oscillations of RCG-measured parameters did not persist after the end of “NPBin” stage.

### 3.10 TCD

Significant (*p* < 0.05) changes in average-over-stage values in response to NPBin were found in CCA PSV, CCA EDV, CCA TAM, MCA EDV. These parameters decreased under NPBin (Table 1), changes did not persist after the end of “NPBin” stage. Changes of CCA EDV and CCA TAM correlated with the level of rarefaction averaged over “NPBin” stage (*p* = 0.003, ρ = −0.55 and *p* = 0.026, ρ = −0.43, respectively), the decrease of CCA EDV and CCA TAM increased with increase in rarefaction. For all of the considered blood flow parameters in CCA and MCA, we found no correlation of the magnitude of changes in parameters with the level of rarefaction during inspiration under NPBin.

**FIGURE 3:**
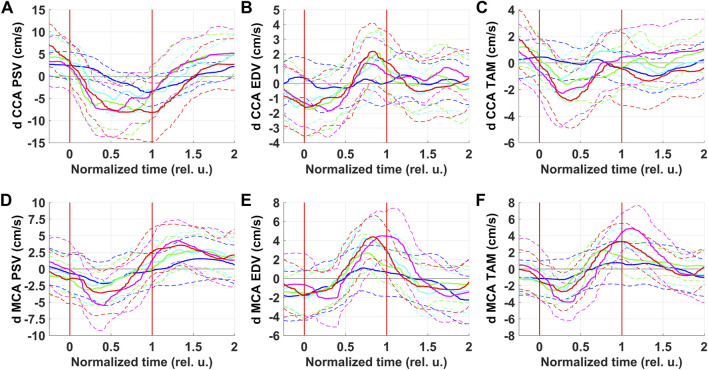
Respiratory oscillations of blood flow velocity parameters in the right common carotid artery (CCA) and in the right middle cerebral artery (MCA). PSV–peak systolic velocity **(A and D)**; EDV–end-diastolic velocity **(B and E)**; TAM–velocity averaged over the current cardiocycle **(C and F)**. Colors correspond to series: blue–NPBin_0 (control series, no additional rarefaction), cyan–NPBin_10 (rarefaction during inspiration set to −10 cmH_2_O), green–NPBin_15 (rarefaction during inspiration set to −15 cmH_2_O), magenta–NPBin_20 (rarefaction during inspiration set to −20 cmH_2_O), red–NPBin_25 (rarefaction during inspiration set to −25 cmH_2_O). Solid curve–changes of parameters at “NPBin” stage of each series averaged over breathing cycles and over the group of volunteers; dashed curves of the same color shows the boundaries of ±SD interval. Vertical lines mark the edges of inspiration. Prefix “d” denotes that the average value of the parameter was subtracted before the calculating of the oscillation (i.e., only the variable component was analyzed).

Figure 3 shows respiratory oscillations of blood flow parameters in CCA and MCA. Respiratory oscillations were observed for all parameters under consideration. In general, measured in CCA and MCA respiratory oscillations of blood flow parameters repeated oscillations of corresponding blood pressure characteristics.

PSV in both CCA and MCA decreased during inspiration and increased during expiration. Under NPBin, peak-to-peak amplitude of oscillations was about 1.5–2 times greater than during free breathing, there was no dependence on the level of rarefaction in NPBin series. Apparently, respiratory oscillations of PSV in both vessels reflected respiratory oscillations of SAP. Respiratory oscillations of MCA TAM were similar to oscillations of MAP.

Respiratory oscillations of EDV in both vessels reflected oscillations of DAP. It is also worth noting that CCA EDV did not oscillate during free breathing; and for MCA EDV, respiratory oscillations were observed during free breathing as well as under NPBin. Most likely, features of blood flow in CCA and MCA influenced: in CCA during diastole, if there are no problems with the aortic valve, blood flow falls to almost zero, zero cannot oscillate; whereas in MCA at the end of diastole blood flow value is far from zero, oscillations are possible.

### 3.11 REG

We found no significant NPBin-induced changes in average-over-stage values of REG parameters (*p* > 0.05, Supplementary Table S1).

REG A decreased during inspiration by 5–10 mOhm. This suggests a decrease in pulse filling of cerebral vessels during inspiration. Under NPBin, the decrease was slightly greater than during free breathing. But curves obtained in different series, including the control one, did not differ within ± SD range. By the end of inspiration, REG A returned to its initial value and did not change during expiration.

Oscillations of REG V/A repeated oscillations of REG A. Respiratory oscillations of REG VO and REG F were not found. Apparently, respiratory oscillations of REG A and REG V/A were completely determined by oscillations of arterial blood inflow, i.e., by oscillations of systemic arterial pressure.

**FIGURE 4:**
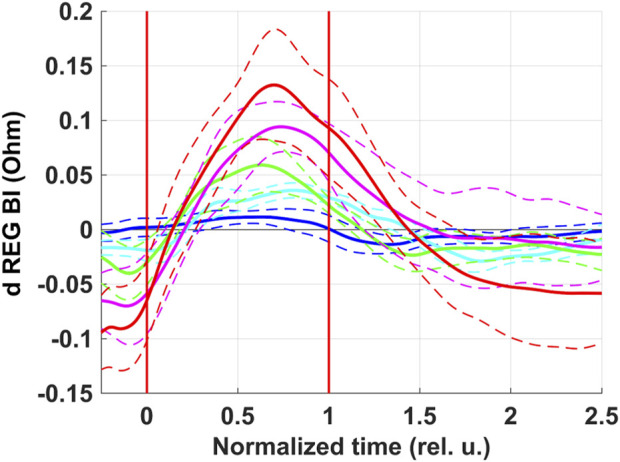
Respiratory oscillations of head electrical impedance. REG BI–basic impedance of REG. Colors correspond to series: blue–NPBin_0 (control series, no additional rarefaction), cyan–NPBin_10 (rarefaction during inspiration set to −10 cmH_2_O), green–NPBin_15 (rarefaction during inspiration set to −15 cmH_2_O), magenta–NPBin_20 (rarefaction during inspiration set to −20 cmH_2_O), red–NPBin_25 (rarefaction during inspiration set to −25 cmH_2_O). Solid curve–changes of parameters at “NPBin” stage of each series averaged over breathing cycles and over the group of volunteers; dashed curves of the same color shows the boundaries of ±SD interval. Vertical lines mark the edges of inspiration. Prefix “d” denotes that the average value of the parameter was subtracted before the calculating of the oscillation (i.e., only the variable component was analyzed).

REG BI was the only parameter for which there was a pronounced dependence of peak-to-peak amplitude of respiratory oscillations on the level of rarefaction during inspiration under NPBin (Figure 4). REG BI rose during inspiration and reached the maximum around the middle of the second half of inspiration. The dependence was practically linear. The maximal increase in REG BI during inspiration under NPBin was approximately 0.2%–0.3% (NPBin_25 series) relative to its mean value (about 45 Ω, Supplementary Table S1). If relative changes in impedance are considered to be equal to relative changes in fluid volume in examined tissue taken with the reverse sign, the maximum decrease in head fluid volume was several milliliters.

### 3.12 LDF

There were no significant differences in changes of average-over-stage LDP values between the control series and series with actual NPBin (*p* > 0.05). In NPBin series, LDP was 4.8 [3.5; 7.3] perf. u. at “before NPBin” stage and 6.1 [3.9; 9.3] perf. u. at “NPBin” stage. At corresponding stages of the control series, LDP value was 6.4 [4.0; 12.3] perf. u. and 7.7 [4.1; 13.4] perf. u., respectively.

During free breathing, LDP decreased slightly during inspiration (Figure 2I) and recovered to initial values during expiration. Under NPBin, starting from the second half of inspiration, there was an increase in LDP, lasting for some time during expiration, then followed by a decrease and return to the initial value. Within ±SD range, we found no dependence of peak-to-peak amplitude of LDP respiratory oscillations on the level of rarefaction.

### 3.13 Capillaroscopy

We found no significant changes in PZ under NPBin (*p* > 0.05). In NPBin series, PZ was 118.6 [107.0; 126.5] μm at “before NPBin” stage and 114.4 [104.2; 124.0] μm at “NPBin” stage. At corresponding stages of the control series, PZ was 113.1 [83.4; 116.9] μm and 107.1 [82.4; 114.8] μm, respectively.

## 4 Discussion

Even in series with rarefaction level of −25 cm of water column, NPBin is a fairly easy-to-endure load (roughly corresponding to the description “moderate” or “somewhat hard”). Changes in breathing pattern observed under NPBin seem to minimize work of respiratory muscles while maintaining minute ventilation almost unchanged. Durations of inspiration and expiration increase, apparently, due to an increase in tidal volume. The increase in V'O_2_ and V'CO_2_ under NPBin, as well as the correlation of the increase with the level of rarefaction during inspiration under NPBin, reflects an increase in work of respiratory muscles, apparently, only partially (too much increase in V'O_2_). One may suppose that a noticeable contribution to the changes in V'O_2_ and V'CO_2_ is made by a change in characteristics of gas exchange in lungs, which reflected, among other things, in washing out of CO_2_ from the body. CO_2_ wash-out was revealed by the decrease in CO_2_ tension in capillary blood and by the transcutaneous data; also, NPBin-induced CO_2_ wash-out was indirectly indicated by the increase in RER.

The absence of clinically significant ECG changes and insignificant changes in concentrations of NT-proANP, NT-proBNP, and BigET-1 in venous blood confirms safety of half-hour use of NPBin with rarefaction during inspiration up to about −30 cmH_2_O.

The observed pattern of NPBin-induced changes in central hemodynamic parameters is in accordance with the previous data (Semenov et al., 2024) and can be explained if we assume that SV is the first to respond to inspiration under NPBin. The decrease in blood pressure and CO in the first half of inspiration is a mechanical consequence of the decrease in SV. In the second half of inspiration, in response to this decrease in parameters, a compensatory reaction of circulatory regulation systems manages to develop. Changes in HR during inspiration under NPBin can be associated with compensatory responses or with the direct mechanical effect of reduced intrathoracic pressure on the heart as well as with the direct mutual influence of structures in central nervous system that regulate breathing and circulation. Using the data obtained, it is impossible to distinguish between neural and mechanical effects on HR.

The decrease in TPR during inspiration, when blood pressure is also decreasing, is of particular interest. One of the reasons may be the “erroneous” functioning of aortic baroreflex. Apparently, intrathoracic pressure during inspiration under NPBin decreases more strongly than arterial pressure. Consequently, despite the decrease in blood pressure, transmural pressure stretching the aortic arch will increase. Under normal conditions, changes in transmural pressure are of the same direction with changes in arterial pressure, the reflex is functioning properly, causing vasodilatation when blood pressure increases and thereby counteracting the increase in arterial pressure. If, under NPBin, transmural pressure during inspiration increases despite the decrease in blood pressure, aortic baroreflex will cause vasodilatation, thereby increasing arterial pressure decrease, whereas carotid baroreflex is causing vasoconstriction. If aortic baroreflex is considered to prevail over carotid baroreflex (Smith et al., 2001), the result will be a decrease in TPR. However, in addition to baroreflexes, other regulatory mechanisms, including local ones, also affect TPR value. Using the data obtained, their effect on TPR cannot be estimated.

The rapid decrease in SV during inspiration can be explained by insufficient blood flow to the left chambers of the heart during diastole caused by stretching of lung vessels: part of venous blood return from the systemic circulation is spent on filling them instead of filling the left ventricle. During expiration, excess blood in the vessels of the lungs is dumped through the left chambers of the heart, leading to an increase in the left ventricle stroke volume. In other words, NPBin enhances the “respiratory pump” by increasing the stretching of the lung vessels. The lack of dependence of peak-to-peak amplitude of respiratory oscillations of circulatory parameters on the level of rarefaction during inspiration under NPBin also supports this assumption. Pulmonary vascular bed has a large compliance, the compliance is determined not only by stretching of vessel wall, but, mainly, by expanding of partially collapsed vessels; in the expanded state, most of them stretch relatively little (Maloney et al., 1970). In a lying position, when majority of pulmonary vessels are expanded, even small additional rarefaction during inspiration is enough to completely stretch pulmonary vessels. Further decrease in intrathoracic pressure cannot stretch them more. An increase in pressure in the respiratory tract leads to suppression of the “respiratory pump” (Skytioti et al., 2018), which also confirms the assumption of the leading role of changes in blood filling of lung vessels in the genesis of oscillations of the left ventricle stroke volume observed under NPBin.

Apparently, the manifestation of hemodynamic effects of NPBin is likely to depend on the position of the body relative to the vector of gravity or on the resulting acceleration vector as the proportion of expanded lung vessels depends on hydrostatic pressure difference (the higher the proportion, the weaker the effect of the “respiratory pump” will be). Effect of body position on NPBin-induced changes in circulatory parameters should be studied in further research.

**FIGURE 5:**
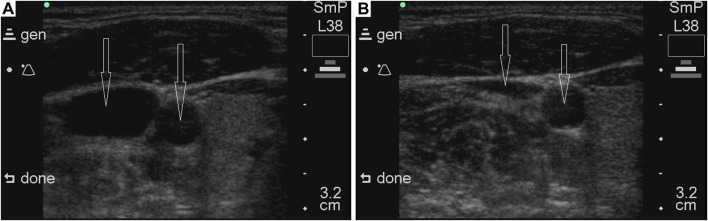
Illustrative ultrasonograms of common carotid artery and internal jugular vein under NPBin of −20 cm of water column. Vessels are marked with arrows; in each of the images, the common carotid artery is on the right. The sonogram on the left **(A)** was obtained during a pause before inspiration, on the right **(B)** – during inspiration under NPBin. The volunteer was lying on his back horizontally. Sonograms were recorded with SonoSite 180 plus (SonoSite, Inc., United States).

Another phenomenon that may limit NPBin effects on circulation is the Starling resistor effect: if the vessel partially collapses, blood flow ceases to depend on pressure in the collapsed part of the vessel. Increasing rarefaction will cause partial collapse of large veins of the systemic circulation, therefore, an increase in venous return to the heart will be limited due to the Starling resistor effect (Guyton et al., 1957). We have observed partial collapse of the internal jugular vein during inspiration under NPBin (Figure 5). If the increase in venous return is limited, NPBin-induced changes in stroke volume and arterial pressure are also limited.

Parameters characterizing hemodynamics in cerebral arteries (REG A, REG V/A, and TCD parameters), in general, repeated NPBin-induced changes in blood pressure in a slightly weakened and distorted manner. Apparently, changes in blood pressure are the prime cause of changes in these parameters observed under NPBin. We did not study the hemodynamics in cerebral veins in detail, but pronounced respiratory oscillations of REG BI with virtually absent respiratory oscillations of REG A indirectly indicate pronounced respiratory oscillations in the volume of cerebral veins under NPBin. Moreover, REG BI is the only one of hemodynamic parameters we studied for which there was almost linear dependence of the magnitude of respiratory oscillations on the level of rarefaction during inspiration under NPBin. Analogous dependence was observed for venous pressure (Yannopoulos et al., 2006a; Yannopoulos et al., 2006b). It should be noted that during each inspiration under NPBin, a decrease in the lumen of the internal jugular vein (Figure 5) is also clearly observed. Similar observations have been presented by Marshall-Goebel with colleagues (Marshall-Goebel et al., 2021). Thus, each inspiration under NPBin is accompanied by a decrease in central venous pressure and an increase in venous outflow from cerebral vascular bed; and the decrease in blood filling of tissues of the head increases in direct proportion to the level of rarefaction in the respiratory tract. Therefore, in order to increase venous outflow from the head under NPBin, it is reasonable to increase the level of rarefaction, but CO_2_ tension in blood or tissues should be monitored to prevent hypocapnia.

By the value of the increase in REG BI it is possible to roughly estimate the value of head blood filling changes and the accompanying decrease in intracranial pressure (ICP). During inspiration under NPBin of −25 cmH_2_O, blood filling of head tissues changed by several mL. Therefore, assuming that all outflow came from intracranial vascular bed, the decrease in ICP was approximately 10 mmHg (Shapiro et al., 1980). During expiration, ICP probably returned to its initial value. Similar results were obtained in studies with invasive ICP measuring (Metzger et al., 2018). Respiratory oscillations of ICP profoundly increased as soon as inspiratory resistance was given, and the oscillations did not persist after the end of NPBin (Yannopoulos et al., 2006a; Yannopoulos et al., 2006b; Metzger et al., 2015).

We found no pronounce changes in microcirculation under NPBin. Observed respiratory oscillations of LDP appear to reflect respiratory oscillations of blood pressure.

To sum up, in healthy individuals under conditions close to rest, the NPBin effect on blood circulation is manifested mainly in respiratory oscillations of central hemodynamic parameters, in main cerebral vessels they are also traced, but weakened. The effect of NPBin on peripheral hemodynamics is weakened even more and virtually absent.

Average values of hemodynamic parameters practically do not change under NPBin, but pronounced respiratory oscillations appear for the parameters. Effects of NPBin on circulation did not persist after the end of “NPBin” stage. For the majority of the parameters, the magnitude of oscillations profoundly exceeds NPBin-induced changes in average values. It can be assumed that the changes in average values are the changes in parameters occurred during inspiration under NPBin, but attenuated proportionally to the portion of inspiration in the duration of the entire respiratory cycle. It follows that: 1) to increase the effect of NPBin on blood circulation, it is necessary to increase the portion of inspiration in the duration of respiratory cycle, but avoiding hyperventilation and hypocapnia (even without significant changes in minute ventilation, NPBin led to some wash-out of CO_2_ from the body); 2) when working with NPBin, the analysis of respiratory oscillations is more informative than the analysis of average values of circulatory parameters; 3) due to the wide individual variation in the magnitude of changes in breathing pattern under NPBin (predominantly, in respiratory rate), NPBin impact on respiratory and circulatory systems may be weaker than expected if the expectations are based only on the level of rarefaction during inspiration.

## 5 Limitations

We should note the observed reactions to NPBin are characteristic of healthy humans under conditions close to rest. Reactions to NPBin in patients or in healthy individuals under extreme conditions may be profoundly different.

The study included seven healthy male volunteers, which was determined based on: a) previous similar physiological studies showing significant effects with comparable sample sizes; b) the comprehensive nature of measurements (simultaneous measurements of multiple physiological parameters); c) the within-subject repeated measures design, which increases statistical power; d) the focus on mechanistic understanding rather than population-level inference. While this sample size was sufficient to detect significant physiological changes and establish clear response patterns, we acknowledge several limitations: 1) the findings may not fully represent the broader population variability; 2) the study included only male participants, limiting generalizability across sexes; 3) statistical power for detecting subtle effects may be limited; 4) individual variation in responses requires careful interpretation. Future studies with larger, more diverse samples would be valuable for confirming these findings across different populations.

## 6 Conclusion

NPBin changes the respiratory pattern: respiratory rate decreases and tidal volume increases. Despite the lack of significant changes in minute ventilation, CO_2_ is washed out of the body. The effect does not persist after the end of NPBin. In the range considered (from −10 cmH_2_O to −25 cmH_2_O), the increase in the level of rarefaction during inspiration under NPBin has little effect on the value of NPBin-induced changes of respiratory and gas exchange parameters.

Average values of hemodynamic parameters practically do not respond to NPBin, but for the majority of parameters the respiratory oscillations appear or intensify under NPBin. Thus, NPBin strengthen the “respiratory pump”. Oscillations of central hemodynamic parameters are the most pronounced, oscillations in peripheral hemodynamics are practically absent. Left ventricle stroke volume during inspiration under NPBin decreases, as well as blood pressure parameters and head blood filling, whereas heart rate increases. Opposite changes are observed during expiration. The decrease in left ventricle stroke volume and central venous pressure during inspiration under NPBin appear to be the major phenomena mediating the effects of NPBin on circulation. For hemodynamic parameters associated with arteries, there is no dependence of the amplitude of oscillations on the level of rarefaction during inspiration under NPBin in the considered range of pressure decrease (from −10 cmH_2_O to −25 cmH_2_O). But the decrease in head blood filling increased almost linearly with increasing rarefaction with no changes in cerebral tissue perfusion. It seems reasonable to use rarefaction during inspiration of no more than 10 cmH_2_O to improve systemic circulation, but greater rarefactions (up to 25 cmH_2_O) to enhance venous outflow from the head.

It is worth noting that the use of an inspiratory rarefaction of 20 or more cmH_2_O is associated with a high load on the respiratory muscles and may be excessive for some patients. In addition, it should be remembered that NPBin in people who have never used respirators, rebreathers, gas masks, or other devices that create inspiratory resistance can sometimes provoke a sharp increase in ventilation, which is almost always a protective psychological-caused reaction. Therefore, whenever possible, patients should be introduced to NPBin before using NPBin, starting with low levels of rarefaction if necessary. The use of NPBin should be accompanied by monitoring the level of CO_2_ in the blood or tissues to prevent hypocapnia. The most convenient for this is the transcutaneous method. We used NPBin for no more than half an hour; there is no information about the effects of longer use. Since inspiration during NPBin is accompanied by an increase in blood filling of the lungs, long-term use, hypothetically, can lead to pulmonary edema. Therefore, the longer use should be accompanied by detailed monitoring of the lung condition.

## Data Availability

The original contributions presented in the study are included in the article/Supplementary Material, further inquiries can be directed to the corresponding author.
