# Supplementary material for: Negative pressure breathing: the response of human respiration and circulation to different levels of rarefaction during inspiration
Source: Front Physiol. 2024 Nov 27;15:1443349. doi: 10.3389/fphys.2024.1443349 (PMC11631904; doi:10.3389/fphys.2024.1443349)
Supplement: Supplementary file 1 [file Table1.docx]

Supplementary Material

**Supplementary Table S1.** Circulatory parameters under NPBin and in the control series.

|  | NPBin series | | | | |
| --- | --- | --- | --- | --- | --- |
|  | Average-over-stage values of parameters | | | Differences in average-over-stage parameter values between stages | |
|  | before NPBin | NPBin | after NPBin | NPBin – before NPBin | after NPBin – before NPBin |
| SAP, mmHg | 121.6 [113.2; 126.4] | 120.7 [111.2; 128.5] | 126.5 [121.1; 131.5] | 0.5 [-1.4; 2.7] | 6.5 [4.2; 8.2] |
| DAP, mmHg | 68.0 [62.8; 72.4] | 68.1 [63.4; 72.3] | 72.9 [67.9; 73.8] | 0.3 [-0.9; 1.8] ** | 4.3 [2.3; 5.5] |
| MAP, mmHg | 86.5 [81.2; 92.3] | 87.1 [80.0; 92.9] | 92.0 [86.4; 95.0] | 0.5 [-0.9; 2.1] * | 5.4 [3.1; 7.1] |
| HR, bpm | 63.9 [59.6; 66.6] | 64.3 [59.9; 69.3] | 60.6 [57.9; 67.5] | 1.4 [-0.8; 3.7] * | -1.9 [-4.7; 0.4] |
| SV, ml | 81.9 [70.4; 104.0] | 86.1 [70.7; 101.2] | 84.4 [70.7; 103.9] | 0.4 [-2.0; 2.1] | 1.0 [-1.3; 4.1] |
| CO, l/min | 5.6 [4.4; 6.3] | 5.9 [4.3; 6.7] | 5.6 [4.4; 6.2] | 0.2 [-0.1; 0.5] | -0.1 [-0.4; 0.2] |
| TPR, mmHg∙s/ml | 0.93 [0.88; 1.10] | 0.94 [0.82; 1.13] | 1.05 [0.91; 1.26] | -0.02 [-0.07; 0.03] * | 0.07 [0.01; 0.14] |
| BRS, ms/mmHg | 22.0 [17.2; 25.1] | 16.7 [14.4; 19.0] | 22.7 [16.0; 27.3] | -3.7 [-7.6; -1.6] + | 0.0 [-2.6; 2.4] |
| RCG A, Ohm | 0.229 [0.185; 0.243] | 0.211 [0.170; 0.221] | 0.198 [0.160; 0.226] | -0.023 [-0.026; -0.011] * | -0.023 [-0.030; -0.011] |
| TT, s | 0.083 [0.076; 0.086] | 0.083 [0.074; 0.088] | 0.083 [0.078; 0.090] | -0.001 [-0.005; 0.001] | 0.002 [0.000; 0.005] |
| ET, s | 0.248 [0.241; 0.262] | 0.249 [0.242; 0.258] | 0.251 [0.242; 0.265] | -0.001 [-0.010; 0.004] | 0.004 [-0.002; 0.010] |
| Tsys, s | 0.330 [0.324; 0.340] | 0.332 [0.319; 0.340] | 0.335 [0.329; 0.346] | -0.004 [-0.013; 0.004] | 0.005 [-0.001; 0.014] |
| Tdia, s | 0.623 [0.577; 0.674] | 0.610 [0.544; 0.662] | 0.645 [0.556; 0.698] | -0.012 [-0.056; 0.011] | 0.026 [-0.019; 0.034] |
| RCG BI, Ohm | 14.2 [13.2; 15.4] | 13.9 [13.0; 15.2] | 13.9 [12.7; 15.1] | -0.2 [-0.5; 0.0] | -0.4 [-0.8; -0.2] |
| REG A, Ohm | 0.088 [0.073; 0.112] | 0.093 [0.074; 0.112] | 0.079 [0.065; 0.099] | 0.000 [-0.005; 0.005] | -0.008 [-0.012; -0.002] |
| REG V/A, % | 57.1 [50.1; 62.5] | 57.1 [52.9; 64.5] | 55.2 [46.8; 60.8] | -0.5 [-2.9; 6.5] | -2.7 [-6.7; 1.2] |
| REG VO, % | 6.9 [-4.2; 17.0] | 10.0 [3.4; 16.9] | 6.6 [-1.3; 11.4] | 2.5 [-2.0; 6.5] | -3.6 [-7.2; -0.3] * |
| REG F, Ohm/s | 0.146 [0.122; 0.182] | 0.153 [0.117; 0.180] | 0.132 [0.111; 0.152] | 0.001 [-0.010; 0.018] | -0.016 [-0.031; -0.004] |
| REG BI, Ohm | 45.9 [40.8; 47.8] | 45.2 [40.6; 47.4] | 44.8 [40.8; 47.3] | -0.3 [-0.7; -0.1] | -0.8 [-1.0; -0.3] |
| CCA PSV, cm/s | 95.2 [79.7; 104.8] | 83.8 [74.9; 97.0] | 89.2 [80.3; 98.4] | -9.4 [-14.4; 1.2] * | -9.2 [-14.6; 3.3] |
| CCA EDV, cm/s | 18.4 [14.9; 21.4] | 14.6 [10.0; 16.9] | 17.4 [15.3; 20.0] | -5.5 [-6.7; -1.5] + | -1.3 [-3.2; 1.8] |
| CCA TAM, cm/s | 38.0 [32.3; 39.1] | 30.9 [27.7; 34.8] | 33.9 [30.8; 37.3] | -6.0 [-7.8; -2.2] * | -3.2 [-5.0; 1.9] |
| MCA PSV, cm/s | 86.6 [77.0; 96.5] | 78.3 [67.4; 96.6] | 84.6 [71.0; 97.7] | -6.3 [-17.1; 1.1] | -2.0 [-7.0; 0.8] |
| MCA EDV, cm/s | 37.2 [32.8; 44.9] | 30.0 [22.8; 42.2] | 36.5 [30.9; 45.5] | -3.2 [-11.5; -0.4] * | -0.1 [-2.8; 3.9] |
| MCA TAM, cm/s | 53.4 [48.5; 63.6] | 44.9 [36.9; 61.7] | 51.8 [45.7; 64.6] | -6.1 [-14.4; -0.5] | -0.4 [-3.4; 2.0] |
|  | Control series | | | | |
|  | Average-over-stage values of parameters | | | Differences in average-over-stage parameter values between stages | |
|  | before NPBin | NPBin | after NPBin | NPBin – before NPBin | after NPBin – before NPBin |
| SAP, mmHg | 118.2 [116.3; 124.7] | 122.0 [119.0; 126.1] | 126.9 [124.4; 133.4] | 1.8 [1.2; 5.0] | 8.7 [5.8; 10.2] |
| DAP, mmHg | 67.8 [64.8; 70.6] | 69.7 [66.9; 72.7] | 72.2 [68.9; 75.1] | 2.1 [1.3; 2.4] | 4.4 [3.8; 6.0] |
| MAP, mmHg | 85.7 [83.7; 89.0] | 88.6 [87.1; 91.6] | 91.8 [90.9; 95.6] | 2.8 [1.7; 3.7] | 6.8 [5.5; 7.4] |
| HR, bpm | 64.5 [60.4; 71.0] | 63.9 [60.0; 70.5] | 61.9 [57.7; 69.7] | -1.1 [-2.4; 0.3] | -2.3 [-2.7; 0.3] |
| SV, ml | 87.6 [76.1; 101.5] | 93.4 [75.0; 99.1] | 87.7 [74.1; 100.1] | -0.9 [-1.9; 1.9] | 0.1 [-1.4; 1.3] |
| CO, l/min | 5.8 [5.2; 6.5] | 5.7 [5.1; 6.6] | 5.7 [5.1; 6.3] | -0.1 [-0.3; 0.0] | -0.1 [-0.3; 0.2] |
| TPR, mmHg∙s/ml | 0.94 [0.85; 1.00] | 0.94 [0.91; 1.06] | 1.02 [0.94; 1.10] | 0.06 [0.01; 0.09] | 0.09 [0.06; 0.13] |
| BRS, ms/mmHg | 19.0 [15.8; 25.8] | 19.5 [14.6; 28.1] | 20.2 [15.7; 30.7] | 1.3 [-0.5; 2.7] | 3.5 [0.1; 4.8] |
| RCG A, Ohm | 0.203 [0.188; 0.249] | 0.185 [0.179; 0.243] | 0.182 [0.173; 0.235] | -0.008 [-0.012; -0.001] | -0.014 [-0.018; -0.010] |
| TT, s | 0.081 [0.078; 0.090] | 0.086 [0.081; 0.089] | 0.088 [0.081; 0.098] | 0.002 [-0.003; 0.005] | 0.003 [-0.001; 0.009] |
| ET, s | 0.254 [0.243; 0.267] | 0.254 [0.245; 0.270] | 0.259 [0.246; 0.281] | 0.001 [0.001; 0.005] | 0.007 [0.004; 0.010] |
| Tsys, s | 0.344 [0.321; 0.356] | 0.339 [0.326; 0.364] | 0.350 [0.335; 0.370] | 0.004 [-0.003; 0.011] | 0.011 [0.005; 0.026] |
| Tdia, s | 0.588 [0.523; 0.652] | 0.597 [0.519; 0.648] | 0.601 [0.518; 0.657] | 0.007 [-0.006; 0.029] | -0.001 [-0.005; 0.028] |
| RCG BI, Ohm | 14.3 [13.2; 15.2] | 14.5 [12.7; 15.1] | 14.3 [12.9; 14.6] | -0.2 [-0.6; 0.0] | -0.4 [-0.7; -0.1] |
| REG A, Ohm | 0.098 [0.075; 0.119] | 0.084 [0.075; 0.120] | 0.079 [0.071; 0.087] | 0.000 [-0.006; 0.001] | -0.012 [-0.021; -0.003] |
| REG V/A, % | 56.0 [50.1; 60.6] | 53.2 [52.5; 61.6] | 54.6 [52.3; 59.5] | 1.5 [-3.0; 4.6] | -0.8 [-3.0; 3.3] |
| REG VO, % | 7.4 [1.4; 17.6] | 9.8 [1.4; 11.9] | 8.1 [3.2; 20.3] | 0.8 [-1.9; 2.4] | 2.1 [-1.3; 8.8] |
| REG F, Ohm/s | 0.159 [0.127; 0.204] | 0.135 [0.127; 0.199] | 0.133 [0.120; 0.143] | -0.001 [-0.016; 0.001] | -0.019 [-0.041; -0.007] |
| REG BI, Ohm | 48.8 [43.7; 51.0] | 48.1 [43.5; 50.5] | 47.7 [43.2; 50.4] | -0.4 [-0.9; -0.3] | -0.9 [-1.2; -0.5] |
| CCA PSV, cm/s | 96.4 [91.0; 100.9] | 92.1 [90.5; 101.3] | 88.8 [81.4; 99.0] | 4.1 [-6.4; 9.0] | -5.9 [-14.7; 1.2] |
| CCA EDV, cm/s | 17.8 [16.4; 22.4] | 18.2 [17.8; 21.6] | 20.3 [15.2; 24.4] | 0.8 [-0.6; 2.1] | 0.5 [-0.6; 2.3] |
| CCA TAM, cm/s | 38.7 [35.0; 42.9] | 36.8 [35.7; 41.9] | 38.0 [33.6; 38.9] | 1.4 [-2.8; 2.3] | -2.9 [-4.1; -1.1] |
| MCA PSV, cm/s | 94.6 [75.6; 115.6] | 92.5 [75.6; 98.9] | 89.6 [79.0; 97.5] | -3.8 [-7.4; -0.5] | -7.4 [-11.3; -1.8] |
| MCA EDV, cm/s | 34.6 [30.6; 50.1] | 35.5 [33.9; 50.3] | 34.0 [32.8; 50.8] | 2.5 [-4.1; 4.5] | 1.6 [-1.7; 3.8] |
| MCA TAM, cm/s | 49.7 [47.2; 74.1] | 53.1 [49.2; 69.6] | 51.3 [48.3; 70.4] | -0.1 [-4.6; 4.0] | -1.4 [-2.7; 2.2] |

The data are presented as M [Q1; Q3], where M is the median, Q1 and Q3 are the first and the third quartiles, values are calculated over the group of volunteers. Signs denote: * - p < 0.05, ** - p < 0.01, + - p < 0.001. P-values were obtained by comparing the same stages of the control series and combined data from NPBin series using the Wilcoxon rank sum test. When calculating the criterion, we used values of changes in parameters relative to their value at "before NPBin" stage. Please refer to "Materials and Methods" section for further details.

**Description of parameters**

**Finometer**

SAP – systolic arterial pressure;

DAP – diastolic arterial pressure;

MAP – mean arterial pressure;

HR – heart rate;

SV – left ventricle stroke volume;

CO – cardiac output;

TPR – total peripheral resistance;

BRS – baroreflex sensitivity.

**Doppler ultrasonography**

CCA PSV – peak systolic velocity (right common carotid artery);

CCA EDV – end-diastolic velocity (right common carotid artery);

CCA TAM – velocity averaged over the current cardiocycle (right common carotid artery);

MCA PSV – peak systolic velocity (right middle cerebral artery);

MCA EDV – end-diastolic velocity (right middle cerebral artery);

MCA TAM – velocity averaged over the current cardiocycle (right middle cerebral artery).

**Rheocardiography (RCG)**

RCG A – amplitude of the systolic wave of RCG signal (vertical distance from the point of the beginning of the RCG pulse wave to its systolic maximum);

TT – period of isovolumetric contraction (period from the ECG R peak to the moment the RCG pulse wave begins);

ET – ventricle ejection period (period from the moment the RCG pulse wave begins to the first minimum of the first derivative of the RCG signal after the systolic maximum);

Tsys – systole duration (the sum of TT and ET in the current cardiocycle);

Tdia – diastole duration (duration of the current RR interval minus Tsys);

RCG BI – basic impedance of RCG (very slowly changing component of the impedance modulus).

**Rheoencephalography (REG)**

We used a procedure proposed by I.V. Sokolova, Kh.Kh. Yarullin et al. (Sokolova et al., 1987)* to analyze REG data. The procedure is based on the assumption that the pulse wave curve of the REG signal (inverted variable component of the impedance modulus) is the sum of two components (arterial wave and venous wave). When analyzing the pulse wave curve of REG signal, five reference points are distinguished (**Supplementary Figure S1**): the beginning of the pulse wave, the systolic maximum (point A), the first inflection point after the systolic maximum (point B), a point at a distance of 4/5 of the duration of the current RR interval from the beginning of the wave (point S), the end of the pulse wave (matches with the beginning of the next pulse wave). Then four indices characterizing cerebral circulation are calculated from the REG signal values at points A, B, S. The list of indices is given below:

REG A (Ohm) – amplitude of the systolic peak of the REG signal, value of the REG signal at point A;

REG V/A (%) – the ratio of the REG signal value at point B to the amplitude of the systolic peak;

REG VO (%) – the ratio of the REG signal value at point S to the amplitude of the systolic peak;

REG F (Ohm/s) – REG F = (A+B)/T, where A is the value of REG signal at point A, B is the value of REG signal at point B, T is the duration of the current RR interval in seconds.

All values of the REG signal are measured from the basic value set by the beginning of the pulse wave in the current cardiocycle.

The value of REG A is used to evaluate the intensity of arterial blood supply to the studied area. According to REG V/A, peripheral resistance of arterial and arteriolar vessels in the studied area is evaluated (the lower the value, the lower the resistance of vessels). The REG VO value is used to evaluate conditions of blood return from the cerebral venous bed. The increase in REG VO corresponds to the increased hindrance to venous return, the decrease corresponds to relief. Unlike other REG indices, REG VO can take negative values. Volumetric blood flow rate is estimated by REG F. The increase in REG F corresponds to an increase in tissue perfusion.

REG BI – basic impedance of REG (very slowly changing component of the impedance modulus).

*Sokolova, I.V., Maksimenko, I.M., Ronkin, M.A, Yarullin, Kh.Kh. (1987). Assessment of the functional state of cerebral vessels by two-component analysis of the rheoencephalogram. [Methodological recommendations of the Ministry of Health of the USSR]. Moscow: Ministry of Health of the USSR. (in Russian)


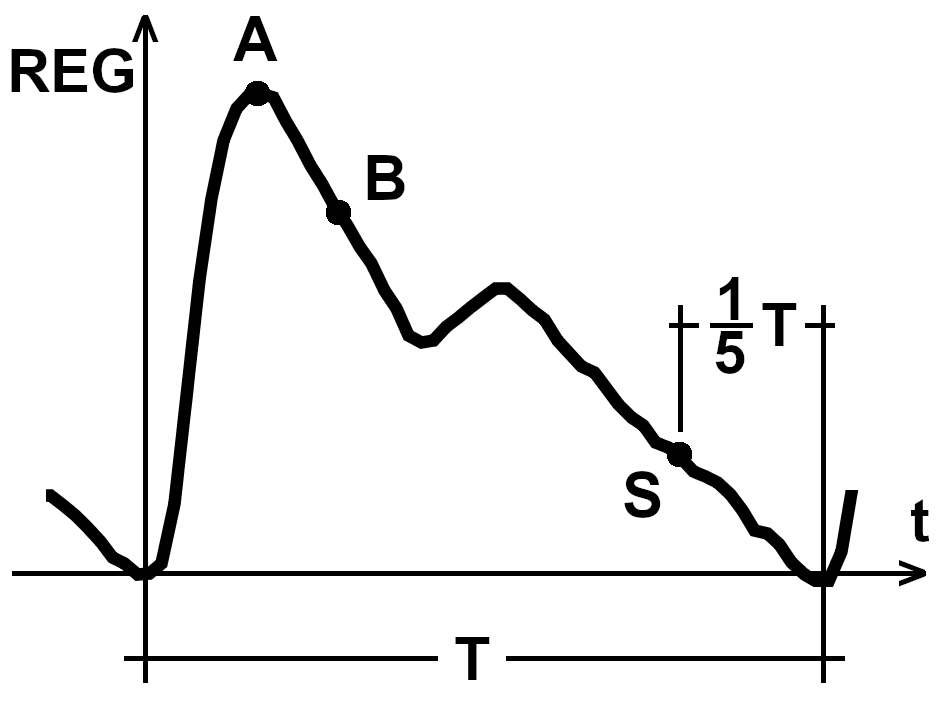


**Supplementary Figure S1.** Marking of the REG pulse wave.
